# The Century-Long Journey of Peptide-Based Drugs

**DOI:** 10.3390/antibiotics13030196

**Published:** 2024-02-20

**Authors:** José R. Almeida

**Affiliations:** 1 Biomolecules Discovery Group, Universidad Regional Amazónica Ikiam, Km 7 Via Muyuna, Tena 150101, Ecuador; rafael.dealmeida@ikiam.edu.ec or j.r.dealmeida@reading.ac.uk; 2 School of Pharmacy, University of Reading, Reading RG6 6UB, UK

The pioneering medical application of peptides as therapeutics began approximately a century ago; however, they remain clinically relevant candidates garnering more attention on the drug development agenda [1,2]. This is reflected in the continuous approval of peptide-based agents by the Food and Drug Administration [3]. For example, last year, five peptides entered the pharmaceutical market after rigorous clinical evaluation [4]. The alphabet of 20 naturally occurring amino acids is the basis of the structural and functional diversity of peptides [5]. These constituent building blocks can be combined in different orders and lengths generating a wide spectrum of short, versatile, and bioactive peptides that are highly valuable in research and drug development [6]. An additional feature is their biochemical flexibility, which allows modifications, amino acid substitutions, or combinations with other biomolecules [7,8]. In general, this flexibility provides a variety of opportunities to modulate the activity, stability, and/or selectivity of peptide candidates [9]. These are some of the key advantages that have made peptides promising structures and key chemical entities for different areas of health and medicine for more than a century, since the first insulin administration. 

Conventionally, peptides have been obtained from natural sources using single or combinatory chromatographic techniques, chemical synthesis, recombinant production, or enzymatic protein hydrolysis [10]. All these strategies are associated with laborious tasks and steps that sometimes result in unsuccessful outcomes evidenced by the lack of the desired activity, low stability in salt or serum conditions, or toxicity leading to significant side effects [11]. However, recently, cutting-edge technologies including computer strategies, such as artificial-intelligence (AI)-based tools [12,13], and cell-free biosynthesis [14,15] have opened exciting horizons for the high-throughput screening, design, rapid manufacturing, and accelerated discovery of medically important and highly active molecules. Despite these advancements, some fundamental questions involving their stability, mode of action, and the fine balance between biological activity and toxicity remain partially understood or unaddressed [16,17]. Even widely studied and hot topics, such as the structure–activity and precise mechanism of action of antimicrobial peptides, remain a challenging puzzle due to their broad chemical space. 

Within the scope of *Antibiotics*, peptides and their derivatives comprise a promising class of line-of-defence agents with favourable and multifaceted properties to respond to the need for effective antimicrobials, such as antiviral, antibacterial, antiparasitic, or antifungal drug templates. In line with this, the Special Issue, “The century-long journey of peptide-based drugs”, for which I served as the Guest Editor, aimed to put together valuable information on antimicrobial peptides and draw attention to the relevant contributions of scientists actively working on the translatability of peptides to address major global issues, such as viral epidemics and antimicrobial resistance. It highlights the increased use of peptides in clinical settings and the milestones in the last century of peptide research that enabled such pharmaceutical progress in real-world environments. Against this background, this Special Issue stands as a guiding light in the collective and multidisciplinary efforts of the scientific community around the world to delve into the biotechnological potential of peptide-based agents and convert them into beneficial therapies and molecular tools for novel discoveries. 

This Special Issue received six manuscripts for consideration, with an acceptance rate of 83.33%. Dr. Domenico Schillaci and Dr. Jean-Marc Sabatier supported different steps of the evaluation and editorial decision making of the submitted documents, serving as academic editors. Finally, this collection presents five important manuscripts that provide a definitive reference and comprehensive source of the latest advances in the utility of peptide-based agents, mainly focused on anti-infective or antimicrobial action. In combination, the two review articles and three original manuscripts published in this collection cover multiple areas of peptide science related to the scope of *Antibiotics*, including (i) anti-infective properties against intracellular pathogens, (ii) the utility and obtention of natural and synthetic cyclic antibacterial peptides, (iii) the mode of action of bagasse-derived peptides, (iv) anti-biofilm and antifungal peptide-based strategies, and (v) the in silico design of dual antiviral and antibacterial peptides. Figure 1 didactically illustrates the interdisciplinary and wide range of topics included in this Special Issue.

Peptide science is a fast-evolving multidisciplinary area that has benefitted from collaborative and team-based efforts. This collection of articles is a clear example of the unlimited boundaries for research teams trying to develop peptide-based drugs. Authors from seven different countries (including Brazil, China, Thailand, Colombia, Ecuador, Chile, and the United Kingdom) have established national and international networks that culminated in the dissemination of novel knowledge and the report of innovative therapeutic modalities for microbial pathogens. The first publication was open-access available on 8 December 2022. 

So far, three of the published articles have been cited, totalling 10 citations in the Scopus database in a short period (assessed on 21 January 2024). This finding evidences the high impact and scope of these publications. The focus, objectives, and key messages of the manuscripts included in this collection are briefly discussed below.

In the first contribution, Cruz and collaborators present a detailed review spotlighting the landscape of peptides able to penetrate cells and induce the death of medically important intracellular pathogens, such as bacteria, viruses and protozoans. This review discusses the complexity of developing cell-penetrating therapeutics that reach the intracellular target in appropriate concentrations, killing them without significant damage to host cells or the emergence of resistance. The multivalent features of cell-penetrating peptides are outlined, and several examples are provided covering their full therapeutic potential, which can improve or even revolutionize the conventional treatment of intracellular pathogens. Finally, in their conclusion, the authors draw attention to two intrinsic issues associated with the biochemical nature of peptides (low stability and selectivity) and reinforce the fundamental role of peptide engineering and structural design approaches to help address these issues. 

In the second contribution, through a comprehensive mini review, Lai et al. highlight the main strengths of cyclic peptide-based antibiotics that may be useful in overcoming the limitations discussed in the first study published in the Special Issue. Initially, the authors contextualise the need for alternative candidate antibiotics in the complex scenario of multidrug resistance. Secondly, a list of the advantages of peptides showing covalently linked cyclic structures is discussed. Then, several natural and man-made cyclic peptide-based antibiotics, including FDA-approved drugs, are presented, which clearly show the progress, clinical use, and therapeutic value of the drug discovery initiatives. In addition, a short overview of strategies employed for cyclic peptide-based antibiotics’ screening and discovery is provided. Taken together, this mini review briefly recompiles the current knowledge of cyclic peptides and delineates the perspectives in the field. 

In the third contribution, Ditsawanon et al. detail the screening of the antibacterial activity of potential bioactive compounds obtained from the enzymatic hydrolysis of agricultural, fishery, and agro-industrial wastes. The findings suggest that bagasse is a rich source of antimicrobial compounds. Motivated by this, the authors characterised and chemically synthesized baggase-derived peptides. A key point in this study was the use of proteomics to investigate the mode of action of peptides, which expands the classical mechanism of AMPs on membranes to the modulation of biosynthetic pathways. In line with the previously published studies in this collection, the authors emphasize the requirement of future stages involving peptide optimization for higher activity and the assessment of cell toxicity. 

In the fourth contribution, Torres et al. explore the antifungal potential of antibacterial peptides from a promising perspective that recognises their broad range of activities. In summary, the authors used a drug discovery strategy that motivates the identification of a new use for bioactive peptides different from the initial therapeutic purpose. In this context, this study investigated the effect of three 20-mer synthetic antibacterial peptides against *Candida* species to explore an additional biomedical application and expand their therapeutic spectrum. The findings evidenced the antibacterial peptides’ characteristics of antibiofilm, anti-Candida, and no toxicity to fibroblasts, which potentially act through membrane rupture and intracellular changes in organelles at a structural level. In conclusion, this research emphasises the importance of a multi-perspective approach for the assessment of the therapeutic potential of peptides for a more holistic view of the possible avenues for drug development. The evaluation of multiple activities or not-reported effects can unlock valuable routes and applications. Finally, the authors highlight a further challenge involving the study of the combination of these 20-mer peptides and conventional treatments for fungal infections. 

In the last contribution, Feijoo-Coronel et al. report their work to combine in silico prediction tools, in vitro screening, and peptide engineering for the discovery of dual-acting peptides exerting both antibacterial and antiviral effects. Starting from the primary structure of the basic subunit of a heterodimeric plant protein (Chenopodin) as an antimicrobial template, the authors designed potential selective peptides guided by computational methods. The approach identified a molecular region (Chen2) within the Chenopodin sequence with antibacterial action. In addition, changes in the amino acid sequence of the Chen1 peptide generated highly active antibacterial peptides (ChenR and ChenW). From a mechanistic point of view, the interaction of Chenopodin-inspired peptides with bacterial membrane lipids seems to be an essential component of their effective action, as suggested by the molecular dynamics and microscopy approach. Overall, this study provides an example of an effective pathway to discover membrane-active and dual-acting peptides driven by computational-based technologies and peptide engineering. However, the stability of Chenopodin-derived peptides in physiological conditions remains a pending task. 

In conclusion, this Special Issue covered the century-long journey of peptide-based antimicrobials, integrating the current state of the art, various developments, and emergent views in peptide research. Together, these articles reiterate peptides’ multidimensional opportunities, revealing new insights into their activities, potentialities, mechanisms of action, and emerging approaches to overcome unresolved tasks and unaddressed limitations. This serves as evidence that this fast-changing field continues to strongly contribute to the development of future therapeutic modalities for antimicrobial infections. In terms of future perspectives, there are sure to be more promising discoveries supported by modern tools that will drive a more accelerated and successful transition of peptide-derived candidates from the pre-clinical stage to clinical settings. Novel technologies, such as AI, have transformative potential to positively impact the new century of this multidisciplinary area, stimulating different approaches for the initial steps of screening that favour the progress to clinically relevant contexts. Another boost for peptide-based drug development may come from peptide conjugation chemistry, which holds strong promise to overcome classical issues and open the therapeutic window. In summary, peptides are expected to continue gaining ground in the pharmaceutical industry as the first line of treatment for different diseases.

## Figures and Tables

**Figure 1:**
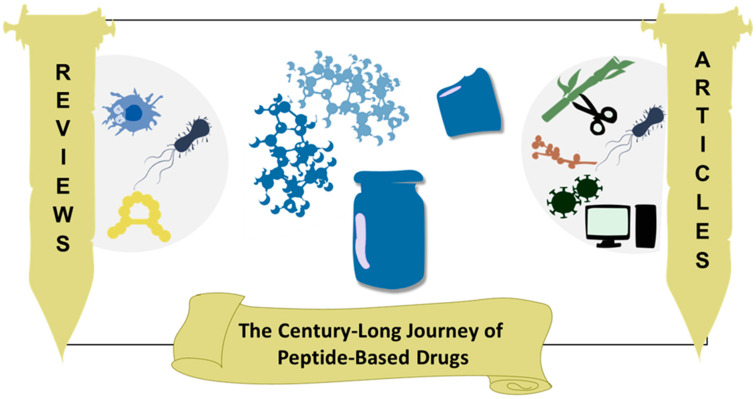
Main topics covered in the Special Issue “The century-long journey of peptide-based drugs”. This collection focused on the antimicrobial applicability of peptide-inspired therapeutics. On the left, the major thematic areas described in the two reviews published in the Special Issue are highlighted. On the right, the focus of the original articles is shown. The peptides in the centre underscore their protagonist roles in drug discovery programs focused on controlling infectious diseases caused by viruses, bacteria, fungi, and parasites.

## Data Availability

No data were generated for this editorial.
